# Financial Literacy, Health Engagement, and Residents’ Health: Evidence from China

**DOI:** 10.3390/ijerph18084202

**Published:** 2021-04-15

**Authors:** Qilong Zheng, Zhen Peng, Shun Ding

**Affiliations:** 1 School of Business, Hubei University, Wuhan 430062, China; zql940358537@gmail.com; 2 School of Medical Business, Guangdong Pharmaceutical University, Zhongshan 528400, China; ds51830890@gmail.com

**Keywords:** financial literacy, health engagement, healthcare, income, health expenditure

## Abstract

This study took residents’ health level as the research object, adopted the perspective of financial literacy, and used the 2014–2018 China Family Panel Studies data to analyze the impact of financial literacy on the residents’ health. The study found that financial literacy could have a significant positive impact on the residents’ health, with long-term effects. Furthermore, it promoted the residents’ health engagement and improved their health through the intermediary effects of income and health expenditure. In addition, the impact of financial literacy on the residents’ health was heterogeneous between urban and rural areas. Compared with the case for rural residents, the improvement of financial literacy significantly improved the health of the urban residents. The outcomes of this research were the exploration of means to improve residents’ health from a new economic perspective, promote residents’ health engagement, and improve residents’ health.

## 1. Introduction

Financial literacy is an important research topic in the field of household finance. Stoney and Stradling first proposed the concept of financial literacy [1]. Due to the development of financial markets, financial literacy has attracted the attention of many large banks, government agencies, and consumers [2]. According to the definition of the Organization for Economic Cooperation and Development (OECD), financial literacy is the ability to obtain financial information, make financial decisions, and realize personal welfare. It includes financial awareness, financial knowledge, financial skills, financial attitudes, and financial behaviors. [3]. Van et al. showed that financial literacy affects personal financial decision-making, and people with low financial literacy are less likely to participate in the stock market [4]. In addition, Chinese and global scholars are also focusing on the research of financial literacy and household financial asset allocation. People with high financial literacy have more diversified investment portfolios [5,6,7], participate in the financial market, and formulate retirement and pension plans. Financial literacy is more likely to promote wealth accumulation [8].

The Concise Report on Consumer Financial Literacy Survey 2019 issued by the People’s Bank of China shows that, compared with 2017, the overall financial literacy of Chinese consumers has improved slightly, their overall financial attitudes have improved, and they have a certain grasp of financial knowledge. However, financial behavior needs to be strengthened. From the perspective of demographic characteristics, the performance of different groups of consumers is quite different. Education, income, region, age, and occupation significantly affect consumers’ financial literacy scores.

Chinese economic development has benefited from the domestic demographic dividend for a long time; therefore, it is particularly important to ensure the health of the labor force. According to the Report on Nutrition and Chronic Disease Status of Chinese Residents 2020, due to the progress of people’s livelihood projects, such as the construction of a healthy China and alleviation of health poverty, Chinese nutrition improvement and chronic disease prevention and control have resulted in advances and significant results, which are mainly reflected in the continued improvement in residents’ physical development, a decrease in nutritional deficiencies, a gradual increase in residents’ health awareness, a steady decrease in the premature mortality rate due to major chronic diseases, a significant reduction in labor losses caused by chronic diseases, and the continued increase in the average life expectancy of residents [9]. However, challenges remain, such as the prevalence of unhealthy lifestyles among residents and the growing problem of overweight and obesity.

In the social sciences, researchers generally believe that the health level of residents is closely related to their socioeconomic status [10]. The income factor in the socioeconomic status has a significant positive relationship with the health level, and the health status of high-income people is usually better because they have access to greater resources [11]. Scholars have discussed the influence of factors such as education and racial discrimination on health [12,13]. However, few Chinese scholars have specifically discussed the impact of residents’ financial literacy on their health. Based on this, this research combined financial literacy with the individual health level, with the aim of exploring whether financial literacy has an impact on an individual’s health level. In addition, financial literacy, as an important form of human capital for individuals, can affect their health by changing income and improving their consumption structure. People with higher financial literacy are more able to make informed financial decisions to obtain higher incomes. In addition, they will also optimize their consumption structure regarding commercial health insurance and health care, obtain better health services, and improve their health.

The empirical steps of this study were as follows: First, we constructed a subjective and objective financial literacy evaluation system, used the coefficient of variation method to assign weights, and calculated the residents’ financial literacy scores. These scores were used to measure residents’ financial literacy. Second, to address the potential issue of multicollinearity between income and health consumption, the correlation between the two factors was tested. If the correlation coefficient was low, we accepted the result and proceeded to the next step. Next, we conducted benchmark regression to test the relationship between residents’ financial literacy and health level, analyzed the long-term effect, conducted a robustness test, and then used an intermediary effect model to explore the impact of financial literacy on health from the perspective of income and health expenditure. In the final section of this paper, we provide conclusions and give policy recommendations.

## 2. Theoretical Basis and Research Hypothesis

### 2.1. The Improvement of Financial Literacy Helps to Improve Residents’ Health

Financial literacy affects residents’ health. The life cycle consumption theory claims that people will plan expenditures over a longer period to achieve the best configuration throughout the life cycle [14]. However, such microeconomic theories usually assume that people are rational and that they have the corresponding financial knowledge, skills, and strong computing power to make and execute plans. As a result, the importance of financial literacy is highlighted. Specifically, financial literacy will have an impact on residents’ credit decisions, savings decisions, consumption decisions, investment decisions, retirement planning, and investment costs. People with higher financial literacy will make more rational decisions to achieve the greatest personal well-being. Furthermore, financial literacy has a positive impact on one’s own health.

Bennett found in his research that for the elderly without Alzheimer’s disease, higher financial literacy leads to better health [15]. Meyer’s research found that people with high financial literacy spend less money to obtain better health services [16]. Financial ability also has a significant and important impact on mental health, where low financial ability aggravates the psychological pressure of related factors [17]. The improvement of financial literacy can improve personal financial abilities, thereby improving the individual’s psychological state, which is conducive to personal health. Therefore, this study believes that the improvement of financial literacy can gradually accumulate advantages in financial knowledge, skills, wealth, psychological resources, and healthy behaviors. People with higher financial literacy will make more rational decisions, improve personal asset allocation, realize personal welfare, and improve personal health.

It has been proved that financial literacy, as a tool for rational decision-making, has a significant inhibitory effect on irrational behaviors such as smoking. People with a high level of financial literacy will reduce the tendency to smoke in the United States and Japan, which shows that improving financial literacy is conducive to individual rational decision-making and improve health [18,19].

Based on the above analysis, this research puts forward the following research hypothesis:

**Hypothesis** **1.** *The improvement of financial literacy will help to improve residents’ health*.

### 2.2. Two Intermediary Paths for Financial Literacy to Improve Residents’ Health

#### 2.2.1. Income Mechanism

As a result of the improvement of financial literacy, residents have greater possibilities to improve their personal socioeconomic status, including increasing their income and obtaining work achievements. Previous studies have suggested that the relationship between financial literacy and wealth is affected by the factor of education, which leads to bias. However, Hu used the path of instrumental variables to identify the impact of the above two factors on wealth accumulation. The results showed that financial literacy has a greater impact on wealth accumulation than education and people with high financial literacy are more likely to accumulate wealth [20]. In addition, He and Li believe that financial literacy has a significant positive effect on personal and family income [21]. Zhang et al. found that the improvement of one’s financial literacy level can significantly improve the performance of family entrepreneurship, thereby obtaining higher entrepreneurial income [22]. Income can significantly improve residents’ health. From the perspective of welfare, groups with higher income are more likely to obtain a better living environment, nutritional status, and medical and health services [23]. From a spiritual perspective, Kaplan’s research found that as income increases, mental health improves [24]. With the increase in income, the living pressure faced by residents is reduced and the initiative to participate in a healthy life is increased. These residents can acquire more health and maintenance knowledge and take action to improve their own health through these health behaviors. Therefore, we believe that financial literacy can affect the residents‘ health through the intermediary variable of income. Scholars have found that people in the United States who are proficient in financial knowledge can reduce their anxiety about old age by realizing asset appreciation [25]. In emerging market Malaysia, the level of financial literacy has a significant impact on individual savings [26].

#### 2.2.2. Health Expenditure Mechanism

People with high financial literacy are more able to rationally allocate financial assets, optimize their consumption structure, and expand their expenditures on health, such as fitness, purchasing health products, and purchasing health insurance. This is conducive to improving their living habits, quality of life, and health. To reduce unexpected expenditures on medical treatment due to health shocks, people with high financial literacy may take the initiative to learn health knowledge to regulate their own health behaviors. They expand their spending on life and health care, such as purchasing health care products, exercising, participating in leisure activities, and consuming the healthy diets, thus achieving the goal of improving one’s own health level and maximizing the utility of their health inputs and outputs. In addition, people with high financial literacy are more likely to buy suitable health insurance. Fan et al. believe that the health effects of commercial health insurance are significant and individuals with commercial insurance have a significant positive effect on promoting personal health [27]. Based on the above analysis, this research proposed the following research hypotheses:

**Hypothesis** **2.** *Income plays an intermediary role between financial literacy and residents’ health. People with better financial literacy will receive more income, thereby improving their health*.

**Hypothesis** **3.** *Health expenditures play an intermediary role between financial literacy and residents’ health. People with better financial literacy will expand their health expenditures, receive better health services, and improve their health*.

**Figure 1:**
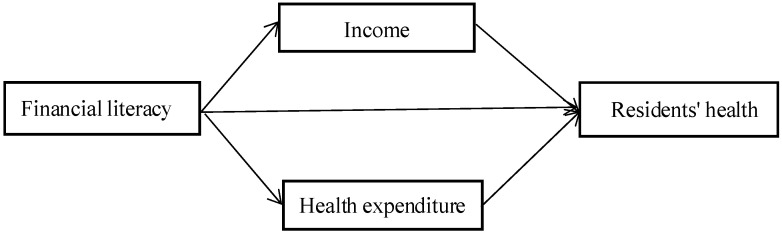
The mechanism of financial literacy affecting residents‘ health.

Based on the above three hypotheses, this study adopted the analytical framework shown in Figure 1 to analyze the relationship between financial literacy and residents’ health.

## 3. Data Sources, Variable Selection, and Descriptive Statistics

### 3.1. Data Sources

The data used in this study were drawn from the “China Family Panel Studies (CFPS),” which was implemented by the Chinese Social Science Survey Center of Peking University. The survey covered 25 provinces (regions, cities), with a target sample size of 16,000 households. In this study, we combined the themes of financial literacy and residents’ health and used the CFPS 2014–2018 survey data, combining the family questionnaire and adult questionnaire to select variables and deleting missing values and samples that did not meet the research requirements. Thus, we obtained a valid sample of 6200 households. The reasons for choosing these data were as follows: The CFPS 2014 questionnaire contains a specialized financial knowledge module, which has a comprehensive range of questions that cover multiple dimensions, such as financial knowledge, financial calculations, and financial attitudes. It includes not only objective issues, such as interest rates, compound interest, inflation, the time value of money, and risk–return pair formation, but also subjective issues, such as formulating financial planning, choosing financial product models, borrowing use, and risk appetite, which can better measure the personal level of financial literacy. In addition, the data in the questionnaire about health level, income, health expenditures, and other related topics are also highly comprehensive, and thus met the needs of this research.

The CFPS sample covers the population of 25 provinces/cities/autonomous regions in China except Hong Kong, Macau, Taiwan, Xinjiang, Qinghai, Inner Mongolia, Ningxia and Hainan. The population of these 25 provinces/cities/autonomous regions accounts for about 95% of the total population of China (excluding Hong Kong China, Macau China, and Taiwan China). The initial target sample size of CFPS is 16,000 households, of which 8000 households are over-sampled from five independent sub-sample frames (called “large provinces”) in Shanghai, Liaoning, Henan, Gansu, and Guangdong, with 1,600 households in each “large province”. Another 8,000 households were drawn from an independent sub-sample frame (called “small provinces”) formed by the other 20 provinces. The CFPS sample is nationally representative in China.

### 3.2. Variable Selection

#### 3.2.1. Health Level

The explained variable of this study was health level. The previous literature usually adopted the method of subjective evaluation. Dummy variables can be used to assign a value of 1 for physical health, and 0 for unhealthy. However, this method may be vague, narrow, and cannot reflect a more specific level of health. Therefore, this study referred to Wang’s practice [28] of using the existing variables in the CFPS data. The health status was divided into seven levels from very poor to very good; that is, the sequential levels from 1 to 7 were set to represent levels of “health,” which was the explained variable.

#### 3.2.2. Financial Literacy

The core explanatory variable of this study was financial literacy. This research measured individual financial literacy in terms of three dimensions, namely, financial knowledge, financial behavior, and financial attitude, in addition to subjective and objective aspects. The comprehensive score of personal financial literacy was calculated using a series of indicators, and financial literacy (fl) was set as the explanatory variable.

#### 3.2.3. Income

Income was the intermediary variable of focus in this study. The total annual income of residents after tax was selected. Because this number is usually large, this study used the natural logarithm to measure it.

#### 3.2.4. Health Expenditure

In the CFPS2014 questionnaire, respondents were asked the related question: “In the past 12 months, how much did your family spend on health care? (including fitness exercise and purchase of related products, equipment, health care products, etc.).” We also took the logarithm of this expenditure to measure the mediating variable of health expenditure.

#### 3.2.5. Control Variable

This study selected variables that are related to personal characteristics, such as age, gender, marital status, and urban or rural location. We selected the levels of medical insurance, local medical quality, exercise frequency, water, fuel, smoking, drinking alcohol, etc., as variables related to health characteristics. These were controlled to ensure the credibility of the empirical results, which are shown in Table 1.

### 3.3. Measurement of Financial Literacy

How financial literacy is measured is still the subject of debate in academia. There are several internationally-used financial literacy measurements: the OECD measurement and Lusardi-Mitchell’s three questions. Studies have shown that unless financial behavior needs to be measured, Lusardi-Mitchell’s three questions are good [29]. However, it is necessary to measure financial behavior in this paper, because financial behavior will directly affect residents’ income and health expenditure, and then affect residents’ health engagement. Therefore, when considering the financial behavior of residents, the OECD measurement is more applicable than the Lusardi-Mitchell’s three questions.

In order to measure financial literacy more accurately, we used a subjective and objective financial literacy measurement system that was constructed by Peng and Zhu, which can better measure the actual level of Chinese consumers’ financial literacy [30]. It follows a part of the OECD measurement. With reference to this system, based on the needs of this paper, 26 secondary indicators were selected from the CFPS2014 financial module questionnaire, and with reference to the practice of Liu and others, the coefficient of variation method was used to set the weights [31]. For the objective questions, the correct answer was assigned a value of 1; otherwise, it was assigned a value of 0. For subjective questions, agree/not difficult/willing were assigned a value of 1; otherwise, it was assigned a value of 0. Then, we calculated the residents’ financial literacy score. The specific indicators and weights are shown in Table 2.

### 3.4. Descriptive Statistics of the Variables

Table 3 shows the descriptive statistics of each variable. It can be seen that the health level of the residents in the CFPS survey sample in 2014 was relatively good, with an average of 5.95. The average score for measuring financial literacy was 0.42, which was less than half of the maximum. In terms of the intermediary variables, the overall health expenditure showed a low level, but the standard deviation was large, indicating that the individual differences were large. The average income level was high, but the standard deviation was also large. In terms of the control variables, the average age of the selected sample was 45 years old (this study only selected respondents over 16 years old), and 52.2% of the survey respondents were female. Most people had some form of medical insurance, accounting for 87% of the total sample. The residents’ exercise situation was not ideal. The average value was only 2.7, which was far below the maximum value of 24. Most people used clean energy and drank healthy water. Residents with smoking and drinking experience also accounted for the majority.

## 4. Empirical Study

### 4.1. Model Setting of the Financial Literacy Measurement

This study explored the impact of financial literacy on the health level of residents. When measuring the health level of the explanatory variable, a numerical value assignment of 1–7 was used, which was discrete. We use the ordered discrete variable of health as the explained variable. If the ordinary multi-value selection model is used, the inherent order of the data will be ignored. For this reason, an ordered probit model (Oprobit model) is required. The model settings were as follows:

(1) $${\mathrm{health}}_{i}=\alpha_{0}+\alpha_{1}\cdot {\mathrm{fl}}_{i}+\sum_{k}\varphi_{1}\cdot {\mathrm{controlvariables}}_{k,i}+\varepsilon_{1,i},\;$$

In Model 1, health is the health level of residents; fl is the financial literacy; controlvariables represents the control variables, which included age, gender, and years of education; *ε* is a random error term. The ordered probit model is non-linear. As such, the coefficients do not represent marginal effects and have no economic meaning; they can only indicate the direction in which the explanatory variables have an impact.

This research also focused on exploring the mechanism of financial literacy on the residents’ health. We believe that financial literacy not only has a direct impact on the residents’ health but also has an in direct impact on the residents’ health by increasing income and health expenditures. Therefore, based on the regression of Model 1, two intermediary variables of income and health expenditure were also added, and the intermediary effect model was used to test the mechanism of financial literacy on the residents’ health. The two intermediary variables were continuous. In order to test the two intermediary mechanisms of financial literacy on the health of residents: income and health expenditure, we used the mediating effect estimation for Models 2–4.Therefore, Ordinary Least Squares (OLS) multiple linear regression andstepwise estimation method were used to explore the impact of financial literacy on income and health expenditures. The form of the model was as follows:

(2) $${\mathrm{health}}_{i}=\gamma_{0}+\gamma_{1}\cdot {\mathrm{fl}}_{i}+\sum_{k}\varphi_{2}\cdot {\mathrm{controlvariables}}_{k,i}+\varepsilon_{2,i},\;$$

(3) $$P_{i}=\beta_{0}+\beta_{1}\cdot {\mathrm{fl}}_{i}+\sum_{k}\varphi_{3}\cdot {\mathrm{controlvariables}}_{k,i}+\varepsilon_{3,i},\;$$

(4) $${\mathrm{health}}_{i}=\lambda_{0}+\lambda_{1}\cdot {\mathrm{fl}}_{i}+\lambda_{2}\cdot P_{i}+\sum_{k}\varphi_{4}\cdot {\mathrm{controlvariables}}_{k,i}+\varepsilon_{4,i},\;$$

where, fl is the core explanatory variable, P is the intermediary variables of income and health expenditures, and control variables represents the control variables. Referring to the previous literature, the coefficients $\gamma_{1}$,$\;\beta_{1}$, $\lambda_{1}$, and $\lambda_{2}$ were stepwise tested according to the steps of the intermediary effect model. This method is called stepwise regression. The Stata 16 software is used to estimate the data of the Models 1–4.

### 4.2. An Empirical Test of Financial Literacy and Residents’ Health Level

Using the ordered probit model to conduct an empirical study of the relationship between financial literacy and residents’ health level with 6200 effective samples, the regression results are shown in Table 4.

In Model 1, only the core explanatory variable of financial literacy was added and it was observed that financial literacy had a significant positive impact on the residents’ health at a significance level of 1%. It can be seen from Model 2 that, after adding the control variables, although the coefficient was smaller, the significant positive impact of financial literacy on the residents’ health still passed the 1% significance test. The two intermediary variables of income and health expenditure were added to Model 3 and it was observed that the coefficient of financial literacy was further reduced. The coefficients of the two intermediary variables were both positive and significant at the 10% level. This shows that income and health expenditure may have had a mediating effect on the level of health. The three models all show that financial literacy had a significant positive impact on the residents’ health, that is, the improvement of financial literacy could improve the residents’ health. Thus, hypothesis 1 was confirmed.

In terms of the control variables, under the same conditions, smoking had a negative but not significant effect on health. The impact of drinking on the residents’ health was negative and significant at the 5% statistical level; this took into account the fact that appropriate drinking is good for health levels and alcoholism causes harm. The difference between urban and rural areas had an impact on the level of health. The coefficient was positive and significant at the level of 1%, indicating that urban residents tended to have a better health level. This may have been because urban residents have access to better resources. Age had a significant negative impact on health. This is in line with general logic. As we age, our physical condition worsens. Exercise had a significant positive effect on health at the 1% significance level, which is also in line with general logic.

### 4.3. Long-Term Effect Test and Heterogeneity Analysis

#### 4.3.1. Long-Term Effect Test

The impact of financial literacy on residents’ health is long-term; that is, financial literacy may not immediately improve residents’ health, and there is a long-term effect. In addition, empirical evidence using only the cross-sectional data of CFPS 2014 may also be biased. Therefore, this study further used the 2016 and 2018 data of the follow-up survey to test the long-term effects. The results are shown in Models 4 and 6 in Table 5, which reports the regression coefficients of the residents’ health status in the 2016 and 2018 follow-up surveys, respectively. The results were similar to those mentioned above, where the regression coefficient was significantly positive at the 1% statistical level, indicating that the positive impact of financial literacy on the residents’ health had a long-term effect. Financial literacy gradually fostered personal well-being in the long term, promoted the improvement of life quality and lifestyle, led to behavioral interventions that were more conducive to health, and improved the residents’ health.

After adding the control variables, financial literacy was found to still have a positive effect on the residents’ health. However, as shown in Models 5 and 7 in Table 5, the regression coefficient of financial literacy in 2016 was only significant at the 5% level. This shows that within a certain period of time, the positive impact of financial literacy on the residents’ health was significant. The regression coefficient of financial literacy in 2018 was not significant. This may have been because the residents’ health was affected by many factors over a relatively large time span, and the effect of financial literacy was affected by many impacts and was no longer significant.

In particular, financial literacy was used as a prevariable, and there was a time difference regarding the health level of residents. Therefore, logically, there was no endogenous problem of mutual cause and effect, and as such, no endogenous treatment was carried out.

#### 4.3.2. Heterogeneity Analysis

Considering that China meets the characteristics of a typical urban–rural dual economic structure, there is a significant gap between urban residents and rural residents in all aspects of life, such as living conditions, income levels, and health services, which may lead to biases in the regression results. To ensure the robustness of the empirical research results, this study further subdivided the sample into urban and rural groups for group regression. We investigated the urban–rural heterogeneity of the impact of financial literacy on the residents’ health. The regression results are shown in Models 8 and 9 in Table 6. Comparing the above two results, it was found that financial literacy had a significant positive impact on the health of urban residents at the 10% statistical level. Although financial literacy had a positive impact on the health of rural residents, it did not pass the significance test. This may have been because, for rural residents, the level of local financial development was low and an improvement of financial literacy may not have been a good incentive for them to participate in the financial market. It is more difficult to improve asset allocation and realize personal well-being, and due to the limitation of living conditions, the health service and medical service systems are not perfect. Thus, expenditure in related areas has not been significantly increased, and there has not been a significant improvement in health. In addition to the heterogeneity between urban and rural areas, differences due to gender may also cause bias in the regression results. Therefore, the sample was divided into males and females for group regression. The regression results are shown in Models 10 and 11 in Table 6. At a significance level of 5%, the improvement of financial literacy had a significant effect on the health of men. Furthermore, it had a more significant positive impact and a greater possibility of improving women’s health. Without considering social and psychological factors, this may have been because women are more sensitive to and perceive greater value in the perception, evaluation, and treatment of long-term mild diseases. The improvement of women’s financial literacy led to higher income and socioeconomic status and, as a result, women paid more attention to their own health.

### 4.4. Robustness Test

#### 4.4.1. Endogenous Test

Financial literacy and the residents’ health are affected by other exogenous factors. The omission of these factors from this study may bias the regression results. Furthermore, financial literacy may also be affected by the residents’ health. Physical health is fundamental to the participation of individuals in the financial market and the improvement of financial literacy. Physical health affects the choices and allocation of residents’ assets. Generally speaking, healthier residents have a greater ability to obtain financial knowledge, participate in the financial market, and exercise financial skills, thus encouraging them to improve their financial literacy. Based on the above two considerations, financial literacy may have endogenous problems due to the omission of variables or the mutual causal influence of the residents’ health level, and the regression results may be biased. In view of this, this study used the instrumental variable method to address the endogenous problem, referring to the method of Zhang and Yin [32]. Thus, we chose the mean value (fl_mean) of the financial literacy level of other people living in the same community or the same village as the instrumental variable. The scientific basis for this approach is that a respondent cannot control his/her neighbors’ financial literacy level, which is strictly exogenous, and neighbors improve their financial literacy via daily life and communication.

This study used the IV-Oprobit model to re-estimate the model. Model 12 in Table 7 reports the instrumental variable regression of the impact of financial literacy on the residents’ health. The results show that the regression results were basically unchanged after the endogenous problem was corrected. Financial literacy still had a significant positive impact on the residents’ health at a statistical level of 1%, which confirmed that the improvement of financial literacy could increase the residents’ possibility of obtaining higher income, optimizing their lifestyles and participating in healthy activities, and improving their own health.

#### 4.4.2. Changing the Metric

Many approaches exist for measuring the variable of financial literacy. Because different methods have different effects on the measurement of this variable, bias may be present in the regression results. Therefore, referring to the method of Lu and Luo [33], this study used the direct summation method to remeasure the financial literacy of the residents. The answers to the 26 secondary indicators mentioned above were assigned a value of 1 according to correct/conformity/willing/will; otherwise, a value of 0 was assigned. These values were directly summed to obtain a new financial literacy variable (fl_1). Then, we undertook the regression again according to the model; the results obtained are shown in Model 13 in Table 7. It was observed that the coefficient of financial literacy was positive and passed the significance test at the 1% statistical level, which was basically consistent with the above regression results. This shows that financial literacy had a significant positive impact on the residents’ health.

#### 4.4.3. Changing the Explained Variable

Furthermore, because many indicators are available to measure the health level, to ensure the robustness of the empirical results, this study selected the indicator “whether hospitalized in the past year” to objectively reflect the health level of the residents. The specific assignment method was as follows: respondents who lived in a hospital during the past year were assigned a value of 1; otherwise, a value of 0 was assigned. The probit model was used for the estimation, and the regression results are shown in Model 14 in Table 7. Financial literacy had a significant negative impact on hospitalization during the past year at a statistical level of 10%, that is, residents with higher financial literacy tended to be less likely to have been hospitalized. This is basically consistent with the previous conclusions stating that financial literacy could enhance the residents’ financial behavior, thus increasing the potential to rationally allocate family assets to achieve personal welfare, improve quality of life and lifestyle, promote healthy engagement, and improve the residents’ health.

## 5. Intermediary Effect Test

### 5.1. The Mediating Effect of Income on the Relationship between Financial Literacy and Health

According to the stepwise regression method described above, the test result of income as an intermediary variable of financial literacy affecting the residents’ health was obtained. The regression is shown in Table 8. In the first step, in Model 15, when the income variable was not added, financial literacy was significant at the statistical level of 1%, which indicated an improvement in the residents’ health. The second step was the regression of income to financial literacy. Model 16 shows that the higher the financial literacy, the more likely residents were to have a higher income. Compared with people with lower financial literacy, people with higher financial literacy were more able to make rational financial decisions to achieve personal well-being and create a higher income. In the third step, Model 17 added income variables on the basis of Model 15. Financial literacy and income had a significant positive impact on the residents’ health at the 1% significance level. These three steps were in line with the test process of the mediating effect model, and all passed the significance test to show that income played a part in the mediating effect of financial literacy on the residents’ health. Among these, the mediation effect accounted for about 22.66% of the total effect, that is, financial literacy could directly affect the residents’ health and could indirectly affect the residents’ health by affecting their income.

### 5.2. The Mediating Effect of Health Expenditure on the Relationship between Financial Literacy and Health Level

From the results of the mediation effect test in Table 9, in model 18, when the health expenditure variable was not added, financial literacy was significant at the 1% statistical level, which was the same as the results in the first column of Table 8. The second step was the regression of health expenditure on financial literacy. Model 19 shows that the higher the financial literacy, the more likely residents were to spend on health. This may have been because people with higher financial literacy improved their consumption structure and paid more attention to improving their quality of life. In the third step, model 20 added health expenditure variables on the basis of model 18. Financial literacy and health expenditure had a significant positive impact on the residents’ health at a statistical level of 1%. The three-step test conformed to the test standard of the mediation effect model, indicating that health expenditure played a part in the mediation effect of financial literacy on the residents’ health. The mediation effect accounted for about 27.72% of the total effect, that is, financial literacy ould not only directly affect residents’ health but also indirectly affected the residents’ health by affecting health expenditures.

## 6. Conclusions

Based on the survey data of the China family tracking surveys in 2014, 2016, and 2018, this study selected 26 subjective and objective indicators and used the coefficient of variation method to obtain the weight of each indicator. In addition, this study measured the level of financial literacy of Chinese residents and used the ordered probit model and OLS multiple linear regression model to empirically analyze the impact of financial literacy on the health level of the residents. On this basis, the study considered influences including gender and urban and rural heterogeneity. Finally, the mediating effect model was used to explore the mechanism of financial literacy on health from the perspectives of income and health consumption.

The empirical results show that: (1) Financial literacy could significantly positively affect health, and individuals with higher financial literacy could improve their health. (2) Financial literacy had a positive long-term effect on the residents’ health. In addition, compared with rural residents, the improvement of financial literacy significantly improved the health of the urban residents. (3) From the perspective of the microinfluence mechanism of financial literacy on the residents’ health, income and health consumption both had a partial mediating effect on the residents’ health.

Highlight the main conclusions of this paper: residents could improve their health by improving their financial literacy, increasing their income, increasing their expenditures on health and health insurance, and improving their quality of life.

In addition to its theoretical significance, emphasizing the impact of financial literacy on residents’ health has significance for policy design in China. First, from the perspective of residents’ financial literacy scores, Chinese residents’ financial literacy levels are still relatively low. The “Concise Report on Consumer Financial Literacy Survey 2019” released by the Central Bank shows that although the overall financial literacy of consumers has increased slightly compared with 2017, the changes in consumer financial literacy should continue to be monitored. Therefore, it is important to improve the financial literacy of Chinese residents. Relevant government departments and financial organizations should further accelerate the popularization of financial knowledge; strengthen the effectiveness of financial education; optimize publicity methods; comprehensively use the Internet, television, and other media; conduct multidimensional publicity in the form of rich media. Second, it is necessary to guide residents to better participate in the financial market such that they can improve their financial literacy level in the practice of the financial market. Finally, the improvement of residents’ health is reflected in the improvement of people’s quality of life. We should further improve the medical and health service system, enhance the service capacity of the health industry, and expand the available types of medical insurance.

Compared with previous studies, this study supplemented two aspects of the existing literature. First, this research innovatively started from the new economic perspective of financial literacy, discussed its impact on the residents’ health, and produced a new path for academic discussion on health. Second, this study placed financial literacy, income, and health expenditure in a unified framework for analysis, and specifically explored how the improvement of financial literacy could improve the residents’ health. Through a detailed analysis of the intermediary path, the mechanism of how financial literacy affected residents’ health was revealed, which provided a basis for the policy formulation of relevant departments.

However, this research is still inadequate. First, limited by the availability of data, the data in this paper is only as of 2018, more recent data should be applied for further empirical research. Second, conducting country comparisons and analyzing the differences of intermediary mechanisms between financial literacy and residents’ health among countries will make sense. Third, more complicated mechanisms may be involved in the effect of financial literacy on residents’ health. These are deserved to be discussed in more depth in the future.

## Figures and Tables

**Table 1:** Description of main variables.

Name of the Variables	Definitions of the Variables	Description of the Variables
health	Health level	Assign values from very poor = 1 to very good = 7
fl	Financial literacy	Calculate the individual scores based on weights
ln(income)	Income	Logarithm of income in the past year
ln(health spending)	Health expenditure	Logarithm of health expenditures in the past year
health insurance	Whether the participant has medical insurance	Has some form of medical insurance = 1, otherwise = 0
medical level	Self-assessment of the local medical level	Thinks that the local medical level is generally very good or good = 1, otherwise = 0
exercise	Number of exercises	Actual exercise frequency
water	Drinking water source	Drinking filtered water/barreled water and other clean water sources = 1, otherwise = 0
fuel	Fuel use	Uses electric energy/natural gas and other clean energy = 1, otherwise = 0
gender	Gender	Male = 1, female = 0
marry	Whether the participant is married	Married = 1, otherwise = 0
urban	Whether the participant is an urban resident	Urban = 1, otherwise = 0
age	Age	Actual age (over 16 years old)
smoke	Smoking status	Smoking in the past month = 1, otherwise = 0
drink	Drinking status	Drinking in the past month = 1, otherwise = 0

**Table 2:** Index system and weights of financial literacy.

Financial Literacy	Index	Definition	Weight
Financial knowledge	Bank fixed interest rate	What is your estimate of the bank’s current 1-year fixed deposit interest rate?	0.0312
	Deposit maturity amount	Assuming you have a one-year fixed deposit of 10,000 yuan and the annual interest rate is 3%, if you do not withdraw in advance, how much money will you have after the deposit matures?	0.0400
	Compound interest calculation	After the deposit in the account in the previous question expires, it will be deposited for 1 year, and the interest rate will remain unchanged. How much money will be in the account after 1 year?	0.0337
	Inflation	If your bank deposit account has a deposit interest rate of 3% per annum and an inflation rate of 5% per annum, how much can you buy with the money in the account in one year?	0.0292
	Time value of money	Assume that Zhang San inherits 100,000 yuan today, and Li Si will inherit 100,000 yuan in 3 years. Which of them has the higher inheritance value?	0.0230
	Symmetry of risk and return	Generally speaking, do high-yield investments have high risks?	0.0131
	Stock investment risk	Generally speaking, is investing in a single stock less risky than investing in a stock fund?	0.0475
	Monetary policy decision maker	Which of the following banks has the function of formulating and implementing monetary policy?	0.0531
	Investment risk	Generally speaking, which of the following assets has the highest risk?	0.0228
	The meaning of buying stocks	What does it mean if you buy stock in a company?	0.0798
	The meaning of a fund	Which of the following descriptions of a fund is correct?	0.0902
	Wealth management product description	Which of the following descriptions of bank wealth management products is correct?	0.0534
	Function of the stock market	Which sentence do you think correctly describes the core function of the stock market?	0.0545
Financial behavior	Shopping affordability	When I buy something, do I consider my affordability?	0.0089
	Pay bills on time	Do I pay bills on time (such as utility bills, credit cards, etc.)?	0.0079
	Pay attention to the financial situation	Am I very concerned about my financial situation (for example, checking my bank account and income and expenditure status often)?	0.0150
	Making long-term finances	Do I make a long-term financial plans?	0.0282
	Financial income and expenditure management	Do I manage my own financial income and expenditure, and also manage my family’s financial income and expenditure?	0.0160
	Consumption patterns	Do I live within my means and consume according to income?	0.0072
	Loan use	Do I use borrowing to maintain a balance of payments?	0.0676
	Charge to an account	Do I have a habit of keeping accounts and record all income and expenses?	0.0441
Financial attitude	The way to choose financial products	When I choose financial products, do I collect product information and compare various products?	0.0212
	Subjective consumer feelings	Is spending money more satisfying than saving?	0.0442
	Subjective tendency	Am I more inclined to live in the present and not think about future things?	0.0373
	Debt multiples	Given your family’s current financial situation, is your family willing to take on more debts?	0.1053
	Difficulty of fundraising	Imagine that in an emergency, you need to raise a sum of 20,000 yuan as soon as possible. How difficult do you think it is to raise this amount of money?	0.0256

**Table 3:** Descriptive statistics of variables.

Variable	Observations	Mean Value	Standard Deviation	Minimum Value	Maximum Value
health	6200	5.95	0.99	1.00	7.00
fl	6200	0.42	0.15	0.00	0.96
ln(health spending)	6200	1.65	3.06	0.00	11.51
ln(income)	6200	10.90	1.18	0.00	14.51
age	6200	45.24	16.06	17.00	95.00
gender	6200	0.48	0.50	0.00	1.00
marriage	6200	0.79	0.41	0.00	1.00
health insurance	6200	0.87	0.34	0.00	1.00
medical level	6200	0.96	0.19	0.00	1.00
exercise	6200	2.70	3.08	0.00	24.00
water	6200	0.95	0.21	0.00	1.00
fuel	6200	0.93	0.25	0.00	1.00
smoke	6200	0.74	0.44	0.00	1.00
drink	6200	0.86	0.35	0.00	1.00

**Table 4:** Ordered probit regression results of financial literacy and the residents’ health level.

Variables	Health		
	Model 1	Model 2	Model 3
fl	0.575 ***	0.318 ***	0.187 *
	(0.0882)	(0.0934)	(0.0962)
ln(income)			0.0337 ***
			(0.0116)
ln(health spending)			0.0234 ***
			(0.00465)
smoke		−0.0305	−0.0306
		(0.0380)	(0.0381)
drink		−0.0936 **	−0.0961 **
		(0.0420)	(0.0421)
urban		0.273 ***	0.260 ***
		(0.0557)	(0.0556)
age		−0.0187 ***	−0.0188 ***
		(0.000989)	(0.000991)
gender		0.0262	0.0269
		(0.0338)	(0.0337)
marry		0.129 ***	0.124 ***
		(0.0361)	(0.0362)
health insurance		0.0761 *	0.0551
		(0.0422)	(0.0422)
medical level		−0.172 **	−0.164 **
		(0.0726)	(0.0728)
exercise		0.0212 ***	0.0197 ***
		(0.00485)	(0.00486)
water		0.0606	0.0371
		(0.0638)	(0.0637)
fuel		0.163 ***	0.138 **
		(0.0574)	(0.0583)
Observations	6200	6200	6200
Pseudo R^2^	0.0025	0.0285	0.0307
chi2 statistics	42.54 ***	468.08 ***	508.48 ***

Note: The robust standard errors are used in the estimation. * represents significance at the 10% significance level; ** represents significance at the 5% significance level; *** represents significance at the 1% significance level. The chi2 statistics are significant at the 1% significance level in Model 1–Model 3, which means these models are significant.

**Table 5:** Results of the long-term effect analysis.

Variables	Model 4	Model 5	Model 6	Model 7
	Health in 2016		Health in 2018	
fl	0.521 ***	0.272 **	0.383 ***	0.166
	(0.113)	(0.117)	(0.123)	(0.127)
gender		0.0677		0.0242
		(0.0431)		(0.0478)
urban16		0.288 ***		0.220 ***
		(0.0671)		(0.0766)
cfps age		−0.0202 ***		−0.0252 ***
		(0.00128)		(0.00137)
marry		0.160 ***		0.0148
		(0.0464)		(0.0540)
medical level		0.102		0.115 *
		(0.0773)		(0.0627)
health insurance		−0.0251		−0.0521
		(0.0558)		(0.0753)
water		−0.0625		0.136
		(0.108)		(0.114)
fuel		0.355 ***		0.137
		(0.0776)		(0.110)
exercise		0.0202 ***		0.0434 ***
		(0.00589)		(0.00650)
smoke		0.00250		0.0250
		(0.0480)		(0.0530)
drink		−0.0323		−0.188 ***
		(0.0533)		(0.0582)
Observations	3966	3966	3224	3224
Pseudo R^2^	0.0020	0.0325	0.0010	0.0382
chi2 statistics	21.18 ***	335.66 ***	9.65 ***	360.65 ***

Note: The robust standard errors are used in the estimation. * represents significance at the 10% significance level; ** represents significance at the 5% significance level; *** represents significance at the 1% significance level. The chi2 statistics are significant at the 1% significance level in Model 4–Model 7, which means these models are significant.

**Table 6:** Results of the heterogeneity analysis.

Variables	Health			
	Model 8	Model 9	Model 10	Model 11
	Urban	Rural	Male	Female
fl	0.286 ***	0.698 **	0.276 **	0.362 ***
	(0.0970)	(0.348)	(0.136)	(0.129)
gender	0.0332	−0.0275		
	(0.0348)	(0.146)		
urban			0.305 ***	0.241 ***
				
smoke	−0.0300	−0.0114	−0.0211	−0.0590
	(0.0392)	(0.159)	(0.0412)	(0.107)
drink	−0.0821 *	−0.236	−0.0839 *	−0.118
	(0.0435)	(0.166)	(0.0447)	(0.124)
age	−0.0189 ***	−0.0145 ***	−0.0172 ***	−0.0204 ***
	(0.00103)	(0.00361)	(0.00150)	(0.00134)
marry	0.120 ***	0.265 *	0.209 ***	0.0520
	(0.0374)	(0.141)	(0.0570)	(0.0473)
health insurance	0.0821 *	−0.0108	0.119 *	0.0423
	(0.0431)	(0.207)	(0.0643)	(0.0556)
medical level	−0.173 **	−0.0933	−0.227 **	−0.114
	(0.0750)	(0.284)	(0.0952)	(0.111)
exercise	0.0215 ***	0.0202	0.0167 **	0.0254 ***
	(0.00500)	(0.0198)	(0.00695)	(0.00679)
water	0.0801	−0.0226	0.0845	0.0348
	(0.0693)	(0.167)	(0.0899)	(0.0902)
fuel	0.106 *	0.459 ***	0.100	0.223 ***
	(0.0624)	(0.148)	(0.0853)	(0.0778)
Observations	5804	396	2976	3224
Pseudo R^2^	0.0273	0.0358	0.0236	0.0340
chi2 statistics	177.05 ***	37.17 ***	91.76 **	133.22 **

Note: The robust standard errors are used in the estimation. * represents significance at the 10% significance level; ** represents significance at the 5% significance level; *** represents significance at the 1% significance level. The chi2 statistics are significant at the 5% significance level in Model 8–Model 11, which means these models are significant.

**Table 7:** Robustness test.

Variables	Model 12	Model 13	Model 14
	Health		Hospital
fl_mean	0.381 ***		
	(0.146)		
fl_1		0.0233 ***	−0.0119 **
		(0.00388)	(0.00593)
age	−0.0186 ***	−0.0182 ***	0.0174 ***
	(0.000978)	(0.000987)	(0.00150)
gender	0.0283	0.0204	−0.0861
	(0.0337)	(0.0338)	(0.0529)
marry	0.130 ***	0.121 ***	0.0660
	(0.0361)	(0.0362)	(0.0580)
health insurance	0.0714 *	0.0634	0.372 ***
	(0.0420)	(0.0422)	(0.0821)
medical level	−0.175 **	−0.180 **	−0.00614
	(0.0727)	(0.0727)	(0.114)
smoke	−0.0254	−0.0373	0.174 ***
	(0.0379)	(0.0380)	(0.0627)
drink	−0.0961 **	−0.0950 **	0.0798
	(0.0419)	(0.0420)	(0.0713)
exercise	0.0220 ***	0.0215 ***	−0.00386
	(0.00484)	(0.00484)	(0.00737)
water	0.0828	0.0632	0.252 **
	(0.0638)	(0.0638)	(0.119)
fuel	0.198 ***	0.175 ***	−0.168 *
	(0.0577)	(0.0577)	(0.0870)
Constant			−2.514 ***
			(0.218)
Observations	6200	6200	6200
Pseudo R^2^	0.0266	0.0314	0.0599
chi2 statistics	468.24 **	542.78 **	256.32 *

Note: The robust standard errors are used in the estimation. * represents significance at the 10% significance level; ** represents significance at the 5% significance level; *** represents significance at the 1% significance level. The chi2 statistics are significant at the 10% significance level in Model 12–Model 14, which means these models are significant.

**Table 8:** The mediating effect of financial literacy on the residents’ health: income.

Variables	Model 15	Model 16	Model 17
	Health	ln(income)	Health
fl	0.318 ***	1.547 ***	0.252 ***
	(0.0934)	(0.101)	(0.0925)
ln(income)			0.0424 ***
			(0.0120)
smoke	−0.0305	−0.0169	−0.0294
	(0.0380)	(0.0400)	(0.0382)
drink	−0.0936 **	−0.0627	−0.0911 **
	(0.0420)	(0.0404)	(0.0427)
urban	0.273 ***	0.182 **	0.266 ***
	(0.0557)	(0.0728)	(0.0559)
age	−0.0187 ***	−0.00483 ***	−0.0185 ***
	(0.000989)	(0.00103)	(0.000940)
gender	0.0262	−0.0268	0.0275
	(0.0338)	(0.0373)	(0.0334)
marry	0.129 ***	0.198 ***	0.120 ***
	(0.0361)	(0.0374)	(0.0348)
health insurance	0.0761 *	0.228 ***	0.0663
	(0.0422)	(0.0454)	(0.0413)
medical level	−0.172 **	−0.0358	−0.171 **
	(0.0726)	(0.0471)	(0.0720)
exercise	0.0212 ***	0.00415	0.0210 ***
	(0.00485)	(0.00509)	(0.00465)
water	0.0606	0.344 ***	0.0461
	(0.0638)	(0.106)	(0.0672)
fuel	0.163 ***	0.467 ***	0.143 **
	(0.0574)	(0.0760)	(0.0575)
Constant		9.287 ***	
		(0.168)	
Observations	6200	6200	6200
Adjusted R^2^	0.0265	0.0831	0.0281
F statistics	45.28 ***	47.79 ***	42.95 ***

Note: The robust standard errors are used in the estimation. * represents significance at the 10% significance level; ** represents significance at the 5% significance level; *** represents significance at the 1% significance level. The F statistics are significant at the 1% significance level in Model 15–Model 17, which means these models are significant.

**Table 9:** The mediating effect of financial literacy on the residents’ health: health expenditure.

Variables	Model 18	Model 19	Model 20
	Health	ln(Health Spending)	Health
fl	0.318 ***	3.530 ***	0.233 **
	(0.0934)	(0.253)	(0.0919)
ln(health spending)			0.0252 ***
			(0.00460)
smoke	−0.0305	0.0380	−0.0315
	(0.0380)	(0.106)	(0.0382)
drink	−0.0936 **	0.198 *	−0.0984 **
	(0.0420)	(0.116)	(0.0427)
urban	0.273 ***	0.316 **	0.266 ***
	(0.0557)	(0.129)	(0.0559)
age	−0.0187 ***	0.00697 ***	−0.0189 ***
	(0.000989)	(0.00270)	(0.000940)
gender	0.0262	0.0207	0.0258
	(0.0338)	(0.0950)	(0.0334)
marry	0.129 ***	−0.0633	0.130 ***
	(0.0361)	(0.0966)	(0.0348)
health insurance	0.0761 *	0.596 ***	0.0619
	(0.0422)	(0.0982)	(0.0413)
medical level	−0.172 **	−0.285	−0.165 **
	(0.0726)	(0.212)	(0.0720)
exercise	0.0212 ***	0.0620 ***	0.0197 ***
	(0.00485)	(0.0131)	(0.00466)
water	0.0606	0.499 ***	0.0477
	(0.0638)	(0.141)	(0.0672)
fuel	0.163 ***	0.469 ***	0.153 ***
	(0.0574)	(0.124)	(0.0572)
Constant		−1.918 ***	
		(0.338)	
Observations	6200	6200	6200
Adjusted R^2^	0.0223	0.0505	0.0283
F statistics	45.28 ***	28.48 ***	44.14 ***

Note: The robust standard errors are used in the estimation. * represents significance at the 10% significance level; ** represents significance at the 5% significance level; *** represents significance at the 1% significance level. The F statistics are significant at the 1% significance level in Model 18–Model 20, which means these models are significant.

## Data Availability

The data that support the findings of this study are available from the corresponding author, upon reasonable request.
